# Optical Coherence Tomography Biomarkers in Diabetic Macular Edema and Their Role in Predicting Visual Outcome After Ranibizumab or Triamcinolone Injection

**DOI:** 10.7759/cureus.113631

**Published:** 2026-07-30

**Authors:** Shanmugasundaram Palanisamy, Priyadarshini Mishra, Bhagabat Nayak, Bruttendu Moharana, Bijnya B Panda, Sucheta Parija, Mathan Kumar Ramasubbu

**Affiliations:** 1 Ophthalmology, All India Institute of Medical Sciences, Bhubaneswar, Bhubaneswar, IND; 2 Pharmacology, All India Institute of Medical Sciences, Bhubaneswar, Bhubaneswar, IND

**Keywords:** biomarkers, diabetic macular edema, disorganization in inner retinal layers, ellipsoid zone integrity, hyperreflective foci, intravitreal injection, oct biomarkers, ranibizumab, triamcinolone

## Abstract

Background

Diabetic macular edema (DME) is the most common cause of defective vision in patients with diabetic retinopathy. With intravitreal injection being a treatment option, different forms of anti-vascular endothelial growth factor (anti-VEGF) and steroid injections are available. In order to choose the effective intravitreal injection based on the retinal changes in optical coherence tomography (OCT) biomarkers, analysis is crucial.

Aim

This study aimed to investigate the changes in OCT biomarkers after intravitreal injection of anti-VEGF (ranibizumab) or steroid (triamcinolone) in DME and to correlate them with visual outcome.

Methods

This prospective observational study included patients diagnosed with diabetic retinopathy with clinically significant macular edema (CSME). Best corrected visual acuity (BCVA) and OCT biomarkers were measured at baseline and after one month of intravitreal injection.

Results

A total of 58 eyes were included. There was a significant change in OCT parameters, central macular thickness (CMT), subretinal fluid (SRF) height, intraretinal cyst height, and hyperreflective foci (HRF), one month after a single intravitreal injection (p<0.05). There was a significant change in BCVA after one injection (p=0.046). Also, a significant correlation was noted between BCVA and CMT (r=-0.455; p<0.001), SRF height (r=-0.438; p=0.001), and intraretinal cyst height (r=-0.540; p<0.001).

Conclusion

OCT biomarkers can be used to monitor therapeutic response and to predict visual outcome after intravitreal injection in DME. The presence of more HRF at baseline might be considered for steroid injection.

## Introduction

Globally, diabetes mellitus (DM) is a major public health problem. Currently, around 6.28% of the world population is affected by type 2 DM, which may increase further by 30% by the year 2045 [1,2]. India, the world's most populous country, has a huge load of DM. It is the fastest-growing disease burden over the last few years, correctly termed as the diabetic epidemic. Retinopathy is a common complication of DM. Around one-third of the type 2 diabetic population develops diabetic retinopathy (DR). Analysis of studies from all over the world reflects the prevalence of DR as 34% and vision-threatening DR as 7%. Macular edema is the most important cause of decreased vision in patients with DR, with a prevalence of 6.81% [3,4].

In DR, several biomarkers can be identified in the blood. Inflammatory and angiogenesis-related biomarkers like C-reactive protein, tumor necrosis factor-α, vascular endothelial growth factor (VEGF), interleukin-6, interleukin-1 beta, interleukin-8, cell adhesion molecules (CAM), and retinol-binding protein have been identified in increased concentration in systemic circulation [5,6]. Although blood samples are easier to collect and provide a larger sample volume, they may not be very specific for retinal pathology. Hence, focus has been shifted more towards local biomarkers such as samples from ocular tissue and fluid, like aqueous humor, vitreous, retina, and tear [7-9]. The key problem is that collecting these samples requires a surgical procedure with a chance of possible complications, except for tear fluid analysis, and such samples are usually of a small volume, which mandates proper collection, storage methods, processing, and analysis to maximize their utility. 

With the advent of spectral domain optical coherence tomography (SD-OCT) with newer techniques, high-resolution images of the retina and choroid can be obtained non-invasively, which facilitates the objective assessment of various quantitative and qualitative parameters. Not only are the measurements highly reproducible, but a substantial degree of inter-observer agreement is also noticed [10,11]. Recently, there has been a rise in interest in these imaging biomarkers because of their non-invasive nature.

Various OCT biomarkers like central macular thickness (CMT), subretinal fluid (SRF) height, hyperreflective foci (HRF), disorganization of retinal inner layer (DRIL), integrity of ellipsoid zone (EZ), external limiting membrane (ELM) integrity, intraretinal cyst height, subfoveal choroidal thickness (SFCT), and choroidal vascularity index can be measured accurately in SD-OCT and are reproducible. These can be used to assess the therapeutic effect and to prognosticate the functional visual outcome [12-16].

## Materials and methods

Aims and objectives

This study aimed to investigate OCT biomarkers in diabetic macular edema (DME) and their changes after intravitreal injections and to correlate these changes with visual outcomes.

Study details

This prospective observational study was conducted at All India Institute of Medical Sciences, Bhubaneswar, in Bhubaneswar, India. Patients diagnosed with type 2 DM within the 35-75-year age group, having moderate to severe non-proliferative DR and early proliferative DR with clinically significant macular edema (CSME) between June 2021 and December 2022, with HbA1c <8%, and with clear media to permit good fundus visualization and proper OCT image acquisition, were included in this study. Patients with type 1 DM, hazy media, history of previous intravitreal injection within six months, vitrectomy, panretinal photocoagulation, presence of epiretinal membrane and macular ischemia, visual acuity worse than 5/60, chronic renal failure of glomerular filtration rate (GFR) <20 ml/min, high-risk proliferative DR including tractional retinal detachment, other microvascular diseases affecting the retina, macula, and optic nerve, high myopia >-6 D, and macular pathologies like age-related macular degeneration were excluded from the study. Patients who developed adverse reactions after intravitreal injection, were lost to follow-up, or required vitrectomy or any other surgical intervention during the study period were excluded as well.

Ethical issues

The procedures followed were in accordance with the ethical standards of the Declaration of Helsinki (2013 revision). Approval was obtained from the Institutional Ethics Committee of All India Institute of Medical Sciences, Bhubaneswar (approval number: IEC/AIIMS BBSR/PG Thesis/2021-22/26).

Methodology

Proper documentation of demographic data and detailed ophthalmological check-up including best corrected visual acuity (BCVA) (using Snellen chart), intraocular pressure (non-contact tonometry), slit lamp examination of the anterior segment, dilated fundoscopy (using 20D and 78D lenses), and SD-OCT imaging was done at baseline and after one month of single intravitreal injection of anti-VEGF (ranibizumab 0.5 mg) or steroid (triamcinolone 4 mg). The choice of injection was based on the patients' affordability. Fundus fluorescein angiography was done only at baseline to rule out macular ischemia.

**Figure 1:**
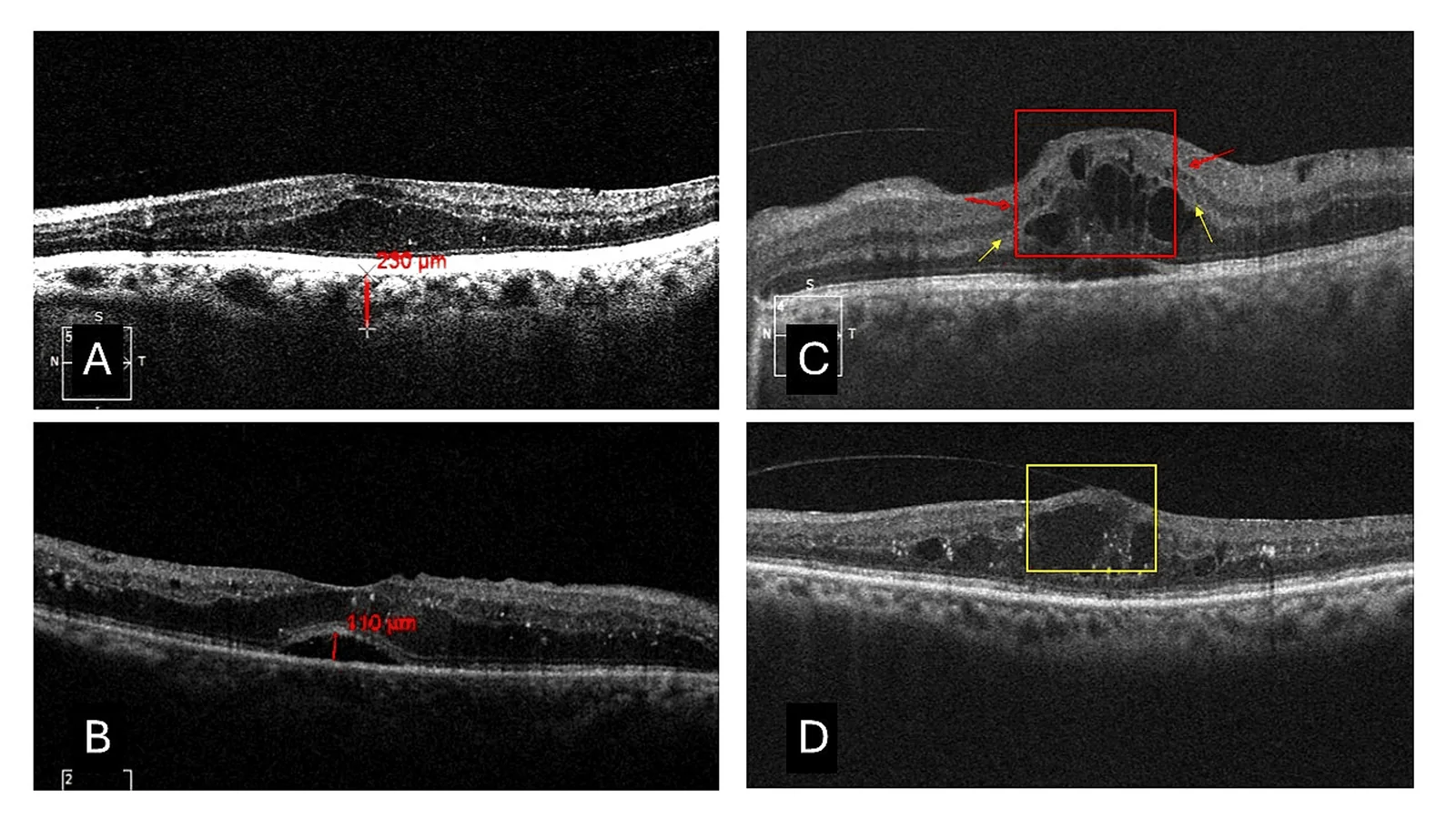
Optical coherence tomography images showing the measurement of parameters (A) Image of HD 5-Line Raster scan with enhanced depth imaging showing the measurement of subfoveal choroidal thickness in µm. (B) Image of HD 5-Line Raster scan showing the measurement of subretinal fluid height in µm. (C) Image of HD 5-Line Raster scan showing absent disorganization of retinal inner layer (red box). Boundaries of ganglion cell layer-inner plexiform complex (red arrows) and inner nuclear layer-outer plexiform layer (yellow arrow) are clearly demarcated. (D) Image of HD 5-Line Raster scan showing disorganization of retinal inner layer (yellow box).

Macular images were acquired using CIRRUS™ HD-OCT 500 (Version 11.0.0.29946; Carl Zeiss Meditec, Inc., Dublin, California, United States) with the standard macular cube 512×128 scanning protocol. CMT measurements were obtained using the built-in automated analysis software of the OCT device. All scans were obtained as fovea-centered. For the evaluation of other parameters, high-definition (HD 5-Line Raster) scans of 6 mm with enhanced depth imaging (EDI) were used. Subfoveal choroidal thickness (SFCT) was measured from the hyperreflective line of the retinal pigment epithelium (RPE)-Bruch's membrane complex to the hyperreflective line of the sclerochoroidal junction (Figure 1A). SRF height was measured at the fovea manually using the built-in caliper perpendicular to the RPE layer (Figure 1B). DRIL was assessed in 1000 μm. In the outer nuclear layer, intraretinal cysts were observed, leading to overall retinal thickening, but the inner retinal layers could still be demarcated, considered DRIL absent (Figure 1C). DRIL was defined as the inability to delineate the inner retinal layer boundaries: any of the boundaries of the ganglion cell layer-inner plexiform (GCL-IPL) complex, inner nuclear layer (INL), and outer plexiform layer (OPL) (Figure 1D). DRIL was graded as follows: DRIL absent, grade 0 (Figure 1C), and DRIL present, grade 1.

**Figure 2:**
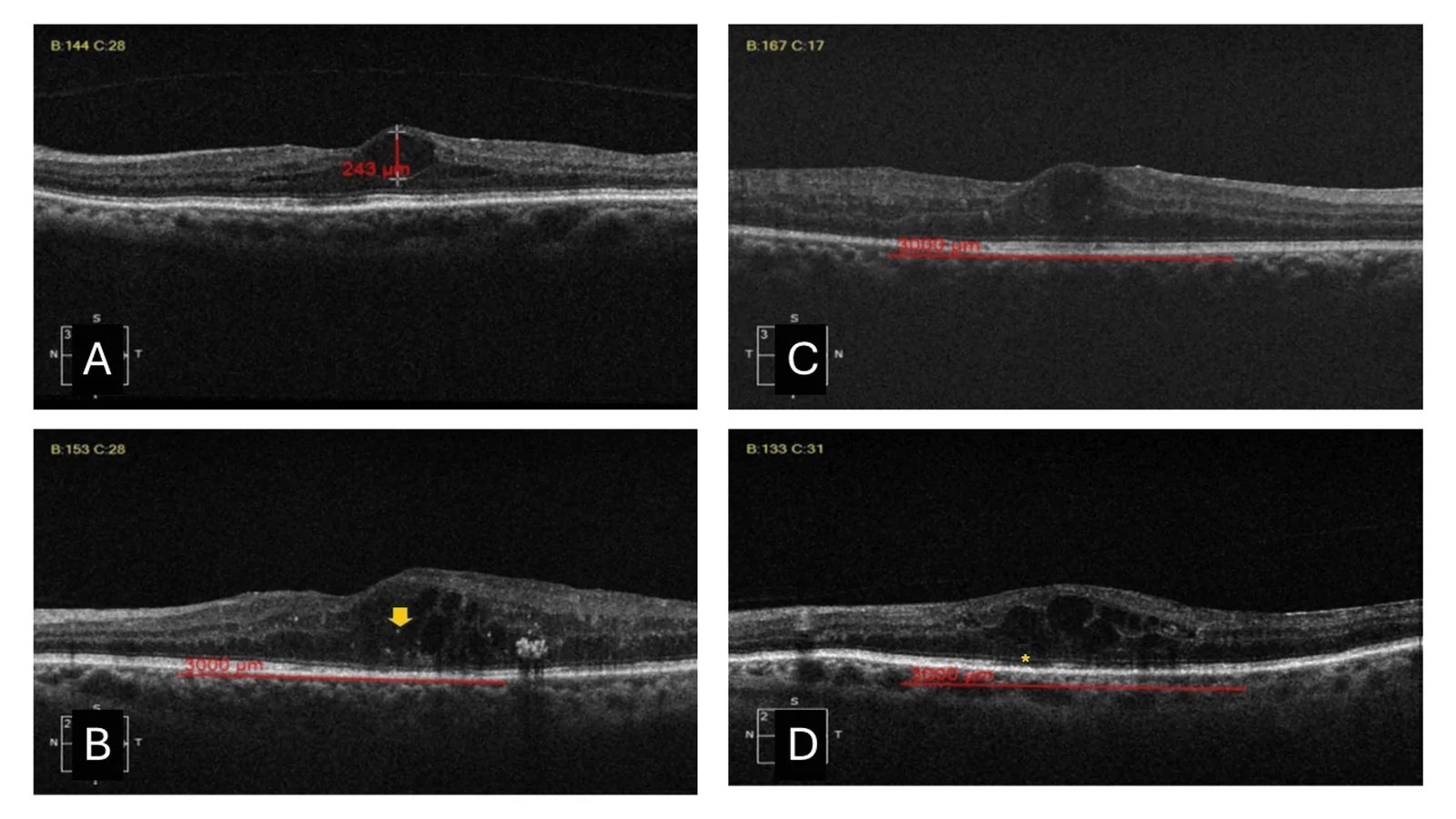
Optical coherence tomography images showing the measurement of parameters (A) Image of HD 5-Line Raster scan showing the measurement of intraretinal cyst height in µm. (B) Image of HD 5-Line Raster scan showing a hyperreflective foci (yellow arrow). (C) Image of HD 5-Line Raster scan showing an intact ellipsoid zone. (D) Image of HD 5-Line Raster scan showing a disrupted ellipsoid zone (yellow asterisk).

Intraretinal cyst height was assessed in cystic spaces present beneath the fovea, the largest of which was taken, measured from the interior face wall in a line, which was perpendicular to the RPE (Figure 2A). HRF and EZ integrity were evaluated within a 1500 μm radius. The number of HRF having high hyperreflectivity like that of the retinal nerve fiber layer (RNFL) with size <30 microns, without backshadowing, and absence of vessels and any lesion in en face imaging were counted manually (Figure 2B). EZ integrity was observed as a second hyperreflective band with RPE-Bruch's hyperreflectivity and was defined and recorded as intact (Figure 2C) and disrupted (Figure 2D).

Statistical analysis

Visual acuity was converted to logMAR values for analysis. Categorical data were represented as frequency and percentage and continuous data as mean and standard deviation. The continuous outcome was checked for normal data distribution using the Shapiro-Wilk test; if it was normally distributed, it was expressed as mean and standard deviation and analyzed using paired and unpaired t-tests. Non-normally distributed data were analyzed using the Mann-Whitney U test. The Wilcoxon signed-rank test was used to compare the mean values of BCVA and OCT parameters between baseline and one month. The correlation between the continuous outcome was analyzed using Spearman's Rho correlation for non-parametric data. The McNemar test was used for paired nominal data analysis of EZ integrity and DRIL. A p-value of less than 0.05 was considered statistically significant. The data were analyzed using IBM SPSS Statistics for Windows, Version 26.0 (IBM Corp., Armonk, New York, United States).

## Results

In total, 58 eyes of 52 patients were included in the analysis, among which 27 patients (29 eyes) received ranibizumab and 25 patients (29 eyes) received triamcinolone. There were 45 male and seven female patients with a mean age of 56.59±8.08. Among these, 28 patients were hypertensives, and two were known chronic kidney disease patients. Chronic kidney disease patients recruited had an estimated GFR interquartile range of 55-80 as the 25th and 75th percentiles. The HbA1c mean is 7.608±1.18.

In this study, there is a significant change in BCVA, CMT, SRF height, intraretinal cyst height, and number of HRF from baseline to one month after an intravitreal injection. SFCT doesn't show much change from baseline. The mean difference of SFCT was very minimal and not significant (p=0.110). In this study, patients who received triamcinolone (Z value: -2.130; p=0.033) showed better BCVA and significant improvement than the ranibizumab group (Z value: -0.787; p=0.431). There is an overall improvement in visual acuity following intravitreal injection (p=0.046), whereas other OCT parameters, such as SFCT, did not show significant changes (p=0.110) in the group analysis as a whole. However, participants who received triamcinolone showed significant changes (Z value: -2.252; p=0.024). The number of HRF was reduced significantly in one month in both groups (p<0.001), ranibizumab (Z value: -2.262; p=0.024) vs. triamcinolone (Z value: -4.461; p<0.001), indicating that triamcinolone has more HRF-reducing property (Table 1).

**Table 1:** Changes in BCVA and OCT biomarkers between baseline and one month after intravitreal injection *Wilcoxon signed-rank test; a p-value of <0.05 is considered significant. OCT: optical coherence tomography; BCVA: best corrected visual acuity; CMT: central macular thickness; SFCT: subfoveal choroidal thickness; SRF: subretinal fluid; HRF: hyperreflective foci

Clinical variables and OCT biomarkers (n=58)	BCVA (logMAR)	CMT (µm)	SFCT (µm)	SRF height (µm)	Intraretinal cyst height (µm)	No. of HRF (counts)
Baseline	0.32±0.25	424.2±134.6	251±30.9	59.24±110.09	190.12±156.79	17.67±11.6
One month	0.29±0.25	337±118.7	250±30.81	12.40±37.66	123.38±155.52	9.57±8.24
Z value	-1.998	-4.913	-1.596	-4.198	-3.244	-4.699
P-value*	0.046	<0.001	0.110	<0.001	0.001	<0.001
Ranibizumab group (n=29)						
Baseline	0.40±0.3	426.83±156.4	247.45±38.46	85.48±141.62	172.34±148.96	18.28±13.01
One month	0.38±0.32	341.14±129.63	249.03±41.44	14.55±43.5	115.45±129.89	11.55±9.71
Z value	-0.787	-3.103	0.000	-3.051	-2.034	-2.262
P-value *	0.431	0.002	1.000	0.002	0.042	0.024
Triamcinolone group (n=29)						
Baseline	0.24±0.14	421.62±111.28	256.45±20.665	33.0±56.4	207.90±164.91	17.07±10.3
One month	0.19±0.06	333.28±109.02	251.0±14.602	10.24±31.3	131.31±179.55	7.59±6.0
Z value	-2.130	-3.904	-2.250	-2.934	-2.190	-4.461
P-value*	0.033	<0.001	0.024	0.003	0.029	<0.001

EZ changes from baseline to one month were insignificant (p=0.500) as a total group and an individual group (Table 2). DRIL showed a significant improvement in one month in patients who received ranibizumab (p=0.039).

**Table 2:** Changes in EZ integrity and DRIL after intravitreal injection *McNemar test (between baseline and one month); a p-value of <0.05 is considered significant. EZ: ellipsoid zone; DRIL: disorganization of retinal inner layer

Parameters	Total group (n=58)	Ranibizumab group (n=29)	Triamcinolone group (n=29)
EZ baseline intact/disrupted	34/24	15/14	14/15
EZ one month intact/disrupted	36/22	17/12	15/14
*P-value (EZ integrity)	0.500	0.500	1.000
DRIL baseline present/absent	41/17	22/7	18/11
DRIL one month present/absent	26/32	14/15	16/13
*P-value (DRIL)	0.001	0.039	0.125

The correlation between OCT parameters and BCVA was assessed in a total of 58 eyes, including subgroups treated with ranibizumab and triamcinolone. CMT showed a significant negative correlation with BCVA in the overall group (r=-0.455; p<0.001), and this correlation was particularly strong in the triamcinolone group (r=-0.641; p<0.001), while it was not significant in the ranibizumab group (r=-0.242; p=0.207). SFCT was also negatively correlated with BCVA in all groups, significantly so across the total group (r=-0.447; p<0.001), ranibizumab group (r=-0.459; p=0.012), and triamcinolone group (r=-0.408; p=0.028). SRF height showed a significant negative correlation in the overall group (r=-0.438; p=0.001) and ranibizumab group (r=-0.532; p=0.003), but not in the triamcinolone group (r=-0.273; p=0.152). Intraretinal cyst height had a strong negative correlation with BCVA overall (r=-0.540; p<0.001) and remained significant in both the ranibizumab subgroup (r=-0.473; p=0.010) and triamcinolone subgroup (r=-0.571; p=0.001). EZ integrity and DRIL were also not significantly correlated with BCVA across all groups. These findings suggest that CMT, SFCT, SRF height, and intraretinal cyst height are important structural parameters associated with visual outcomes, particularly in eyes treated with triamcinolone (Table 3).

**Table 3:** Correlation analysis of OCT parameters and BCVA *Spearman's Rho correlation analysis; a p-value of <0.05 is considered significant. OCT: optical coherence tomography; BCVA: best corrected visual acuity; CMT: central macular thickness; SFCT: subfoveal choroidal thickness; SRF: subretinal fluid; HRF: hyperreflective foci; EZ: ellipsoid zone; DRIL: disorganization of retinal inner layer

OCT parameters	BCVA in the total no. of eyes (n=58)		BCVA in the ranibizumab group (n=29)		BCVA in the triamcinolone group (n=29)	
	Correlation coefficient (r)	*P-value	Correlation coefficient (r)	*P-value	Correlation coefficient (r)	*P-value
CMT (µm)	-0.455	<0.001	-0.242	0.207	-0.641	<0.001
SFCT (µm)	-0.447	<0.001	-0.459	0.012	-0.408	0.028
SRF height (µm)	-0.438	0.001	-0.532	0.003	-0.273	0.152
Intraretinal cyst height (µm)	-0.540	<0.001	-0.473	0.010	-0.571	0.001
HRF (counts)	+0.067	0.618	+0.180	0.351	-0.050	0.796
EZ integrity	-0.015	0.913	-0.033	0.863	-0.307	0.296
DRIL	-0.100	0.457	-0.041	0.833	-0.530	0.062

## Discussion

The purpose of the study was to assess the visual outcome predictor among the different OCT biomarkers after intravitreal injection in DME patients. In our study, we recruited 58 eyes of 52 diabetic patients with DR with a male-to-female ratio of 6.4:1.

Here, the important key factor is visual improvement after the resolution of macular edema and other inflammatory changes in the retina due to DR, following the therapy of intravitreal injections. In our study, patients received intravitreal anti-VEGF (ranibizumab 0.5 mg) and steroid (triamcinolone 4 mg) in two different groups. After one month, visual acuity and OCT parameters were assessed.

As a result, there is a significant improvement in BCVA and OCT parameters like CMT, SRF height, and HRF. In studies by Paccola et al. [17] and Massin et al. [18], it was mentioned that triamcinolone was more effective in reducing macular thickness than ranibizumab. Still, in our study, the mean difference of CMT in the ranibizumab and triamcinolone groups (85.69 µm vs. 88.34 µm) was equally effective. But this depends on the baseline data and glycemic control status of the patient. There is no macular thinning noticed in our study. Karst et al. [19] have reported cases of macular thinning in patients treated with ranibizumab injections.

The mean baseline SFCT was 247.45±38.46 µm in the ranibizumab group and 256.45±20.665 µm in the triamcinolone group. There was no significant change noticed in SFCT after intravitreal injection; the mean difference was very small in both groups. Previous studies by Schocket et al. [20] and Kim et al. [5] reported that the choroidal vasculature plays a vital role in maintaining retinal health and facilitating the clearance of inflammatory mediators, thereby promoting faster recovery from retinal damage.

The mean baseline SRF height was 85.48±141.62 µm in the ranibizumab group and 33.0±56.4 µm in the triamcinolone group. After one month of intravitreal injection, SRF height was 14.55±43.5 µm in the ranibizumab group and 10.24±31.3 µm in the triamcinolone group. This change is clinically significant in both groups. In our study, sensory neural detachment (SND) was noticed in 12 eyes in the ranibizumab group and 15 eyes in the triamcinolone group. Both groups showed good resolution of SRF, but this was significant in the ranibizumab group (p=0.002) and triamcinolone group (p=0.003). These SRF reductions had a negative correlation with visual acuity and were significant in the ranibizumab group (r=-0.532; p=0.003). This concludes that ranibizumab is better than triamcinolone in terms of SRF reduction. However, studies by Hwang et al. [13] and Bonfiglio et al. [14] reported that SND eyes showed poor visual outcome and poor response to ranibizumab. Zur et al. [8] mentioned in their study that steroids respond well to SND.

The mean baseline intraretinal cyst height in our study was 172.34±148.96 µm in the ranibizumab group and 207.90±164.91 µm in the triamcinolone group. After one month of intravitreal injection, the intraretinal cyst height was 115.45±129.89 µm in the ranibizumab group and 131.31±179.55 µm in the triamcinolone group. This change was clinically significant in the ranibizumab group (p=0.042). These intraretinal cysts damage the tissues in the plexiform layers, which were good predictors of visual outcome reported in a study by Pelosini et al. [9]. These intraretinal cysts responded well to ranibizumab according to a study by Reznicek et al. [12]. The presence of hyperreflective dots indicates inflammation of the retina, which responds well to steroids, as concluded in studies by Zur et al. [8], Hwang et al. [13], and Bonfiglio et al. [14]. In our study, both groups showed a significant reduction in HRF, but triamcinolone showed more efficacy (Z value: -4.461; p<0.001) than the ranibizumab group. The EZ represents the photoreceptor layer's health. Hence, EZ plays a vital role in visual prognosis following intravitreal injection. In our study, there is no significant change in EZ status from baseline to one month. Zur et al. [8] and Koc et al. [15] have mentioned in their studies that EZ integrity contributes to visual improvement. DRIL damages the retinal supporting cells and leads to damage and derangement of retinal layers. Borrelli et al. [16] had reported in their study that DRIL is a negative predictor of visual outcome.

In our study, BCVA, CMT, SFCT, SRF height, intraretinal cyst height, and HRF were significantly improved at one month. In a subgroup analysis, CMT, SRF height, intraretinal cyst height, HRF, and DRIL were significantly improved in the ranibizumab group, whereas in the triamcinolone group, CMT, SFCT, SRF height, HRF, and intraretinal cyst height were significantly improved. OCT parameters CMT, SFCT, SRF height, and intraretinal cyst height correlated significantly with visual outcome. When a subgroup analysis was done, in the ranibizumab group, SFCT, SRF height, and intraretinal cyst height correlated significantly. HRF demonstrated a greater response to intravitreal steroid injection than to ranibizumab, supporting their predominantly inflammatory origin, consistent with findings from previous studies. The presence of more HRF at baseline may be an indication for intravitreal steroid injection.

The disparity of results in other parameters in our study and other studies may be due to the small sample size. Another fact is that we did all the analysis after a single dose of intravitreal injection, whereas in most other studies, analysis was done at least after three months. Also, there is a need for the standardization of the measurement of these parameters so that all studies can be compared more effectively.

The strength of our study is that we studied the changes in seven OCT biomarkers prospectively in DME patients treated with anti-VEGF and steroids. The limitations of our study are that the sample size is small and the follow-up period was short.

## Conclusions

In our study, the OCT parameters CMT, SRF height, and intraretinal cyst height showed significant change at one month after a single-dose intravitreal injection of ranibizumab or triamcinolone and correlated with the change in visual acuity. HRF particularly responded more to steroid injection than to ranibizumab. To conclude, these OCT biomarkers can be used to see the therapeutic response and to predict the visual outcome after intravitreal injection in DME. The presence of more HRF at baseline may be an indication for triamcinolone injection.
